# Fitness profile and training of Special Operation Forces: a comparison with sports athletes

**DOI:** 10.3389/fspor.2025.1594714

**Published:** 2025-05-21

**Authors:** Alain Dössegger, Thomas Gsponer, Martin Flück, Christian Protte, Thomas Wyss, Eric Häusler, Markus Gerber, Oliver Faude

**Affiliations:** ^1^ Swiss Federal Institute of Sport Magglingen, Magglingen, Switzerland; ^2^ Department of Sport, Exercise and Health, University of Basel, Basel, Switzerland; ^3^ Stadl Partners, Münsingen, Switzerland; ^4^ Department of Endocrinology, Metabolism and Cardiovascular System, University of Fribourg, Fribourg, Switzerland; ^5^ Zentrum f. Nieren-, Hochdruck- und Stoffwechselerkrankungen, Hannover, Germany; ^6^ Percoms AG, St. Gallen, Switzerland

**Keywords:** tactical athletes, Special Operations Forces (SOF), physical performance, fitness assessment, fitness profile, training

## Abstract

**Introduction:**

Tactical athletes of Special Operations Forces personnel face diverse, high-risk demands—from explosive power actions and maximal strength tasks to prolonged endurance efforts and rapid decision making under stress. This study aimed to develop a multidimensional fitness profile for these tactical athletes by defining Critical Success Factors and translating them into measurable performance indicators, then comparing Swiss Special Operations Forces operators and candidates with sports athletes.

**Method:**

In a cross sectional, observational design, 262 male participants completed a tailored battery of laboratory and field tests: 69 Special Operations Forces operators, 175 Special Operations Forces candidates, and 18 athletes from disciplines such as decathlon, Thai boxing, wrestling, and ice hockey. Practitioner interviews established key mission critical factors, which were operationalized into tests of reaction time, power, strength, severe intensity muscular and cardiopulmonary work capacity, and aerobic endurance. To evaluate group and unit effects, we compared two Bayesian linear regression models: a baseline model (m₀) and a model (m₁) that included group as a fixed factor. Evidence for a group effect was quantified by the Bayes Factor and Probability of direction, where >90% is considered equal to an alpha level of 0.05, thus a non-negligible effect.

**Results:**

Weekly training volume averaged 8.5 h for operators, 6.9 h for candidates, and 9.5 h for athletes. SOF operators and candidates demonstrated fitness levels comparable to sports athletes. Mean jumping distance was 2.43 m, relative hand-grip strength 1.51 kg/kg body mass, deadlift with 100 kg was 20 repetitions, and maximal oxygen uptake measured 54 ml/kg/min. Candidates recorded faster computer based reaction times than operators, while operators outperformed candidates and athletes in a close-quarters combat simulation. Both candidates and operators completed fewer 100 kg deadlift repetitions compared to the mean of the athletes.

**Discussion:**

Swiss Special Operations Forces operators and candidates demonstrate fitness profiles on par with sports athletes across multiple domains, validating their designation as tactical athletes. The normative values and test battery offer a tool for identifying individual strengths and weaknesses, guiding targeted training programs, and informing selection and readiness assessments. Future research should explore longitudinal interventions and predictive models of operational performance.

## 1 Introduction

Special Operation Forces (SOF) play an increasingly important role in warfare and homeland security. SOF need to prevail by campaigning prior to, or in the absence of, armed conflicts (1). Their tasks during missions include activities such as protection, reconnaissance patrol, close protection, intervention and access technique for static, dynamic and evolving situations. Additionally, they undergo training in emergency medical response, infantry tactics, shooting, close combat, rope and helicopter techniques, and survival skills, to name just a few (2).

SOF operators—commonly referred to as “Tactical Athletes” (TAs)—are individuals whose professional roles combine the peak physical demands of elite sport with the unpredictable, mission-specific requirements of military and law-enforcement operations (3, 4). TAs face far broader and more variable demands than traditional sport athletes. Their physical and psychological requirements shift with each training phase, mission profile, and unit specialization, while the operational environment—characterized by uncertainty, volatility, and prolonged stress—calls for fitness profiles that are both multidimensional and adaptable (5–7).

Comprehensive evaluation of SOF performance therefore necessitates consideration of multiple variables of physical fitness and beyond—including cognitive ability, personality traits, and adaptability (8). Decades of U.S. Department of Defense–sponsored research (9) have highlighted eight core competencies for high-risk operators: Stress tolerance, adaptability, cooperation, physical fitness, stamina, judgment, motivation, and initiative. Still, reliably measuring these remains challenging given the inaccessibility of Tier 1 populations and the complexity of their operational contexts (e.g., sniper vs. breacher vs. medic). Likewise, Eisinger et al. (10) observed that evidence-based motor profiles for Special Forces soldiers were lacking and instead derived key sports-motor components—such as coordinative abilities, strength endurance, aerobic endurance, and reaction speed—through expert interviews and ranking questionnaires, demonstrating the value of practitioner-driven task analysis.

In many high-performance domains—business, sport, even military leadership—Key Performance Indicators (KPIs) have been adopted to provide objective, actionable measures of progress toward critical goals. In sport science, KPIs have been shown to inform training decisions, chart performance trends, and predict competitive outcomes (11). However, for KPIs to be meaningful in a tactical context, they must be anchored in Critical Success Factors (CSFs), the essential capabilities required for mission effectiveness. Originating in corporate strategy (12) and adapted in military settings (8, 13), CSF-KPI logic links essential requirements with measurable performance metrics. To structure performance assessment, we thus conducted interviews with experienced SOF instructors and operators to define CSFs. These CSFs were then analyzed and translated into KPIs, such as maximal strength or anaerobic power, which in turn were operationalized into a coherent battery of field and laboratory tests.

We chose sport athletes as a reference group because they provide performance benchmarks across multiple fitness domains, allowing us to contextualize SOF results against the upper limits of human physical capacity. Where possible, we further align our fitness response variables with published data on Olympic-level athletes and international SOF units to situate our findings within both elite sport and tactical populations.

### 1.1 Aim

The present study defines a multidimensional fitness profile for TAs. We want to describe the fitness profile that reflects KPI of TA, and present evidence for differences or non-differences in fitness profiles of TA, candidates for SOF (CSOF), and sports athletes (SA). This study provides initial reference values for fitness response variables. Further, it compares the fitness levels of Swiss SOF operators and candidates to those of national and international high-level sports athletes.

## 2 Materials and methods

### 2.1 Study population

Study participants were recruited from the military, police, and from sport. In total, 262 male participants—69 SOF operators (TAs), 175 SOF candidates (CSOF), and a convenience sample of 18 sports athletes (SA)—actively training at the time, agreed to participate.

The SA cohort represented a broad spectrum of performance calibers: international-level professionals (including a Swiss National Team floorball player, professional wrestler, and national water-polo athlete), national-level semi-professionals (ice-hockey My League and recently retired National League players; Thai-boxing and boxing competitors in national title bouts), and competitive regional-level athletes (decathletes, a middle-distance runner, a triathlete, a regional powerlifter and CrossFit athlete, and volleyball players at both retired national and amateur-league levels). SOF operators and candidates were enlisted from both professional and militia units across police SOF in multiple cantons and military SOF formations [Military Police SOF, Grenadiers, Parachute Reconnaissance, and a VIP close protection/support unit (SOFA)]. Supplementary Table S1 (Supplementary file) provides an overview of all compared groups.

Prior to participation, military and police commands—or those responsible for the respective units—provided potential SOF participants with a “participant information flyer” and a “patient information/informed consent” document detailing the study's aims, procedures, benefits, and risks. Participants were free to decline without consequence and were assured that individual test results would remain confidential and not disclosed to recruiting officers. Written informed consent was collected by the commands, and each SOF participant received a unique study ID (letter + 5-digit number) to protect identity. Sports athletes were directly recruited by the main author based on discipline and availability and underwent the same informed-consent process and anonymization. Participants were included only if they were currently free from any injury and/or illness, free from intake of any medication likely to disturb heart rate, did not take medication such as antihistamines, antidepressants, etc., were free from any cardiovascular or chronic lung disease, were not diagnosed for long COVID after Sars-CoV-2 infection, passed risk stratification and met minimal criteria of the recruiting authorities in any field other than physical or cognitive. The study was performed in accordance with current ethical guidelines (Declaration of Helsinki, as revised in 2013) and was approved by the Human Research Ethics Committee of the Canton of Berne (approval n° 2022-00767), as well as the research committee of the Swiss Armed Forces (Kompetenzentrum Militär- und Katastrophenmedizin).

### 2.2 Development of KPI and test operationalization

Following the identification of practitioner-derived CSFs via interviews, an interdisciplinary expert panel translated each CSF into one or more sport-science–based Key Performance Indicators. Drawing on established principles of exercise physiology and biomechanics, we mapped specific CSFs to performance constructs, e.g.,:

• Explosive movements and combat actions (e.g., jump/Land in full gear, punch and kick hard) were framed as power (generating impulse, explosiveness).
• Lifting and dragging tasks (e.g., lift and hold 50 kg, enter through a window in the 1st floor after pulling oneself up, drag 100 kg) were framed as measures of maximal and repeated strength.
• Prolonged load carriage and fatigue resistance (e.g., hike with 40 kg for 7 h, run or climb several stairs in gear, hand fight, recover quickly) translated into severe-intensity muscular and cardiopulmonary work and aerobic endurance.
• Perceptual-cognitive demands and emotional regulation (e.g., detect threats, scan environment, up-regulate aggression and calm down quickly) were quantified via executive functioning, reaction-time and reactive stress-tolerance paradigms, combining cognitive load with physiological stress markers.

For each KPI, a panel, consisting of sport scientists, a sports physician, psychologists with extensive SOF selection experience, a physiologist, and a molecular biologist, selected at least two complementary tests to ensure robustness. Explosive power paired the standing long jump with a novel striking-power dynamometer task; maximal strength paired hand-grip dynamometry with a modified midthigh pull; strength endurance incorporated weighted pull-ups and deadlift repetitions; and perceptual-cognitive capacity combined computerized dual-task tests with a novel close-quarters battle reaction-time task using Fitlight® sensors. Aerobic endurance was measured by ergospirometry. All strength and endurance tests were performed with participants wearing plate carriers to mimic operational load carriage.

To validate face validity and operational relevance, we conducted an online questionnaire with 34 active military and police SOF operators, asking them to rate each test on a 5-point Likert scale (1 = “Do not agree at all” to 5 = “Fully agree”) in response to the statement: “The test is relevant to my job; it measures the skills I need on the job.” Ratings of 4 (“Somewhat agree”) or 5 (“Fully agree”) were classified as “accepted.” Acceptance rates ranged from 62% for the Standing Long Jump and Isometric Deadlift Test to 97% for the FX/Fitlight® reaction test. Operators' comments—such as “Very balanced test with many relevant exercises related to my profession”—further supported the practical relevance of the fitness battery (14).

This scientifically grounded, yet innovative test battery—balancing ecological and face validity, normative comparability, and logistical feasibility—provides a comprehensive profile of TAs performance and underpins the comparative analyses presented in this study.

**Figure 1:**
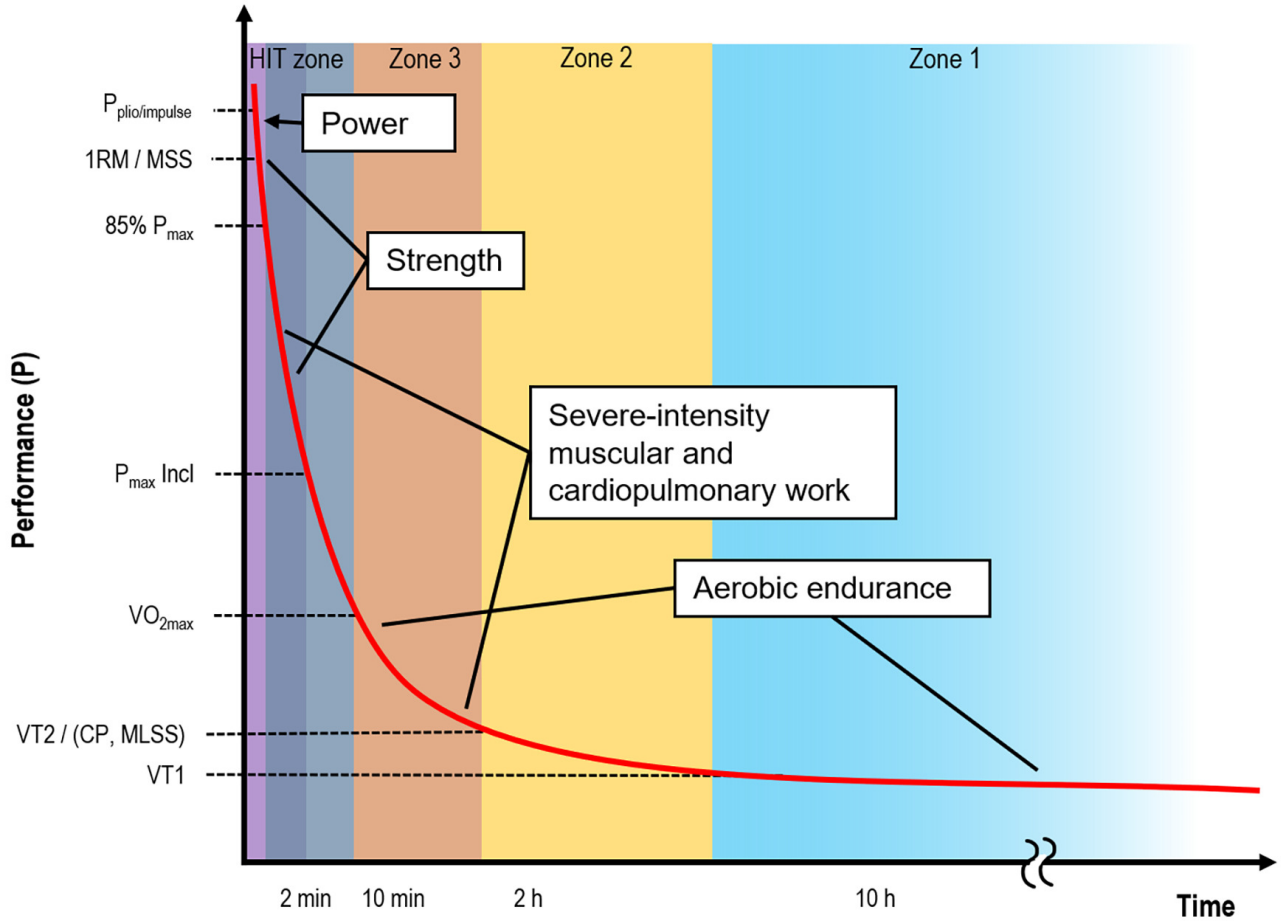
Idealized relationship (hyperbolic red line) between physical performance (P, e.g., Watt, Joule) and duration of maximal performance (Time). The higher the performance or energy expenditure, the shorter the duration in which the effort can be performed (adapted from and Poole, Burnley (15), Hill (16)). SOF physical fitness demands cover almost the entire continuum from maximal power to aerobic endurance. In addition to the fitness tests along the red line, reaction time was measured as one aspect of executive functions. Furthermore, questionnaires were filled out to capture training habits and physical and mental strain and recovery. P = performance; Pplio/impulse = Force production pliometrically (excentrically)/impulse (i.e., jump, strike); 1RM = One-Repetition Maximum; MSS = Maximal Sprinting Speed; VO_2max_ = maximal oxygen consumption; VT = Ventilatory Threshold; CP = Critical Power; MLSS = Maximal Lactate Steady-State. Zones refer to training zones 1 (basic endurance, recovery zone), 2 (zone 2 endurance, zone of lactate accumulation), 3 (development zone, heavy specific endurance), HIT = High intensity training (long and short intervals, repeated sprints).

The physical fitness tests dimensions covered most of the spectrum from ultra-short power production to aerobic endurance (see Figure 1). The curve of maximal human performance over time follows a hyperbolic function (15, 16), representing the maximal available work capacity (Wʹ). This curve provides insight into the “finite amount of work that can be completed during exercise prior to the attainment of the limit of tolerance” (17). Figure 1 illustrates the theoretically maximal performance that can be sustained over time, along with the fitness dimensions assessed in this study. Table 1 summarizes each fitness test alongside its KPI dimension and an example CSF construct.

**Table 1:** Overview of fitness response variables, their associated KPI dimensions, and illustrative critical success factors.

Fitness response variable	Fitness dimension (KPI)	CSF construct (example)
Mean reaction time FX/Fitlight® (RT_FX_)	Reaction time	Shoot-and-move transitions, suppress reactive impulses, analyze situations, detect threats, be situational aware, scan environment
Median reaction time DT (RT_DT_)		React adequately very quickly to stimuli
Standing longjump (SLJ)	Power	Jump-and-land in full gear, repetitive sprinting
Upper body striking power (SP_u_)		Punch hard (effect on target), end fights quickly
Lower body striking power (SP_l_)		Kick hard (effect on target), accelerate
Power to lift/pull (DeadliftPower_1RM_, PullupPower_1RM_)		Enter through a window in the 1st floor
Relative hand grip strength (HGS_rel_)	Strength	Lift and hold 50 kg multiple times
IDT peak isometric force [N] (IDT_peakf_)		Lift and drag 100 kg, carry equipment
IDT peak kg relative to body mass (IDT_rel_)		
One repetition maximum deadlift (Deadlift_1RM_)		Lift 120 kg (operator in full gear) multiple times
One repetition maximum pullup (Pullup_1RM_)		Pull up a balcony in gear
Repetitions deadlift (Deadlifts_rep_)	Severe-intensity muscular and cardiopulmonary work	Lift heavy equipment, carry heavy loads
Repetitions weighted pullups (wPullups_rep_)		Climb ladders in full gear, climb mountains in gear
Maximal blood lactate accumulation (Lac_max_)		Grappling and hand to hand combat for several minutes, maintain fighting attitude, be determinated
Performance at maximal inclination [W/kg body mass] (P_max_ Incl)		Walk several stairs in full equipment
$\dot{V}O_{2\max}$	Aerobic endurance	Patrol in full gear, swim and dive
Performance [W/kg body mass] at $\dot{V}O_{2\max}$ (P_VO_2max__)		Be fatigue resistant
Performance [W/kg body mass] at VT2 (P_VT2_)		Run in full gear
Percentage of the inclination at VT2 (%I_VT2_)		
Percentage of the inclination at VT1 (%I_VT1_)		March with 40 kg for 7 h without food
Uphill running economy [L/min at 4°] (URe4°)		Take high ground position with equipment
HR recovery 60 s after test stop (HRR_60_)		Recover quickly after intense activity

KPI, key performance indicator; CSF, critical success factor; RT_FX_, mean reaction time via Fitlight® close-quarters combat system; RT_DT_, median reaction time in the determination test; SLJ, standing long jump; SP_u_, upper-body striking power; SP_l_, lower-body striking power; DeadliftPower_1RM_, peak mechanical power per kg at estimated deadlift 1RM; PullupPower_1RM_, peak mechanical power per kg at estimated pull-up 1RM; HGS_rel_, hand-grip strength relative to body mass; IDT_peakf_, peak isometric force in the isometric deadlift test; IDT_rel_, isometric deadlift force relative to body mass; deadlift_1RM,_ estimated one-repetition maximum deadlift; Pullup_1RM_, estimated one-repetition maximum pull-up; deadlifts_re*p*_, repetitions of 100 kg deadlifts; wPullups_rep_, weighted pull-up repetitions with 12.6 kg vest; Lac_max_, maximal capillary blood lactate accumulation; P_max_ Incl, power output at maximal treadmill incline; $\dot{V}O_{2\max}$, maximal oxygen uptake; P_VO₂max_, power output at $\dot{V}O_{2\max}$; P_VT2_, power output at second ventilatory threshold; %I_VT2_, percent of maximal incline at VT₂; %I_VT1_, percent of maximal incline at VT1; URe4°, uphill running economy at 4° incline; HRR_60,_ heart-rate recovery 60 s post-exercise.

### 2.3 Testing procedures and fitness response variables

Study participants were measured either in their military base or at an arsenal in Switzerland, running in a fixed order through a test battery using essentially the same equipment. After confirming their study-ID, and oral explanation of the test procedures, body height was measured using a stadiometer (Model 214; Seca GmbH, Hamburg, Germany). The Frankfurt plane was used as reference for head positioning. Body mass was measured using a calibrated digital balance (Model 877; Seca GmbH, Hamburg, Germany), and Body Mass Index (BMI) was calculated. Following the computerized stress-tolerance test (see below), participants completed a standardized warm-up: 7–10 min on a Technogym Synchro crosstrainer (Technogym, Cesena, Italy), followed by dynamic functional gymnastics drills to mobilize and activate the major muscle groups.

#### 2.3.1 Reaction time

Participants performed the complex multiple-stimuli reaction test “Determinations Test” (DT) to assess reactive stress tolerance using the Vienna Test System (Schuhfried GmbH, Moedling, Austria), selected to assess continuous, sustained rapid and varied reactions to rapidly changing stimuli. The DT demonstrates excellent internal consistency (*r* = 0.98–0.99) and has established validity in distinguishing clinical and normative groups (18). It has been applied in police officers (19), elite athletes (20), and is routinely used in the psychological assessment of Swiss professional SOF candidates and operators. By providing objective, validated metrics of reactive stress tolerance under pressure, the DT captures the sustained rapid-decision demands critical to SOF missions.

RT to visual stimuli with a simulation handgun (RT_FX_), including visual scanning, decision making and suppressing reaction to stimuli in a close quarters combat-like situation, was measured using the Fitlight® system (FITLIGHT Sports Corp., Aurora, Canada) and a Glock 17 T FX handgun (GLOCK Ges.m.b.H., Deutsch-Wagram, Austria) in TA and CSOF. Three Fitlight® pods were placed 3.5 m from the center of a 50 cm square taped to the floor, at 1.5 m height, with a 60° angle between each pod. Participants were instructed to put out the light by either tapping or shooting at the pod if the lights went on, depending on the color. Blue lights were decoys that had to be ignored. The Fitlight® pods were programmed in the advanced settings of the Fitlight® app to light up in five different colors for a maximum of 5 s or until tapped or shot, with the pods' proximity and touch sensors turned on. Delay between the lights decreased between each of the 18 stages. Participants had one magazine with 15 rounds of marking Simunition® with a 0.4 g plastic projectile (General Dynamics Ordnance and Tactical Systems Canada Inc., Repentigny, Canada) for each of the two attempts. Average RT and missed shots were noted, and RT for each pod was recorded within the Fitlight® proprietary application. SA did not perform the DT and the RT_FX_ due to time restrictions and a lack of familiarity with weapon handling.

#### 2.3.2 Power

Power was measured using the standing longjump (SLJ) and a striking power measuring device. For the SLJ, participants jumped from the floor onto 7 cm thick gym mats, and the distance from the starting line to the proximal heel was recorded to the nearest cm in accordance to the Swiss Army fitness test (21, 22). The SLJ is a reliable and valid measure of lower body power (23). Upper body striking power (SP_u_) was measured by elbow and hammer fist strikes, lower body power (SP_l_) by knee strikes and low kicks with the dominant side. After some familiarization strikes, we recorded the best striking power of around 7–10 strikes, excluding values that were unreasonably high. The impact pad was individually adjusted to body height. Strikes were measured with the PowerKube^TM^ (Strike Research Limited, Norwich, England), a device equipped with speed transducers, which captures two SI-based components, namely peak power, a short-lived spike (lasting millionths of a second) measured in watts, reflecting the speed-dependent force at impact, and kinetic energy, measured in joules, representing the sustained energy transfer that contributes to a strike's penetrative effect—enhanced by the athlete's center-of-gravity movement during impact. The PowerKube™ algorithm then computes a compound “Impact Power” score. According to the manufacturer, the proprietary Franklin (f)—named after inventor Kevin Franklin—combines instantaneous power and sustained kinetic energy into a single, standardized impact metric. The inventor positions Franklin as the de facto standard for human-impact measurement in sport and academia, with reported use by elite organizations such as the UFC.

#### 2.3.3 Strength

Maximal isometric force was operationalized as handgrip strength (HGS) and isometric deadlift pull force, measured by the isometric deadlift test (IDT). HGS of both hands was measured using a hydraulic hand dynamometer (SH5001®, Saehan Corporation, Changwon, South Korea) with the participants in the seated position, elbow at 90°, handle adjusted to the second or third position according to their preference. After familiarizing with the instrument, participants should apply maximum HGS for 3–5 s. The procedure was performed three times with each hand alternately, with an interval of 1 min between each measurement. Relative HGS (HGS_rel_) was calculated by summing up the highest value (kg) per hand, divided by body mass (24).

For IDT, a 40 cm long handle bar was attached to a strain gauge (Transmetra ZW1.0, Flurlingen, Switzerland) and to a 100 × 40 cm wooden board to measure maximal peak force of participants pulling the handle with wrist straps in a semi-sumo stance with both bare feet on the board. The height of the handle bar was set at 40 cm to imitate the Rautek rescue grip height of a seated patient. After a warm up with an Olympic weight lifting bar, participants were instructed to familiarize with the proper lifting position on the board, with the feet turned 15° outwards, bar between the knees, shoulders back, flat back, before pulling the bar slowly and in a controlled manner. The peak value (kg) of three attempts was recorded and normalized to body mass; peak force (Newton) was calculated using the formula from a validation study (25). In addition, Pullups_1RM_ and Deadlifts_1RM_ were calculated using the Brzycki (26) formula (Equation 1, Table 2), for 8 repetitions of weighted pullups and deadlifts (see below) and under, the Epley (29) formula (Equation 2) for 10 repetitions and over, and a linear interpolation of the formulae for 9 repetitions. This hybrid approach is grounded in empirical validation (26, 29–31) and empirical findings from the Strength Level one-rep-max calculator (https://www.strengthlevel.com). Relative maximal power (J/s per kg body mass) was calculated with the respective estimated 1RM (Equations 3, 4), lifting height and pull-up displacement were approximated from pilot measurements in 10 subjects, yielding mean values of one-quarter body height and arm length minus 33 cm, respectively, to parameterize these calculations.

**Table 2:** Equations used to calculate 1RM, rel. power, and mechanical work on the inclined treadmill.

Equation	No
1RM=load[kg]/(1.0278+0.0278⋅repetitions) (26)	(1)
1RM=load[kg]⋅(1+0.0333⋅repetitions) (29)	(2)
$\mathrm{PullupPowe}r_{1\mathrm{RM}}[W/\mathrm{kg}\;\mathrm{body}\;\mathrm{mass}]\;=\;(\left[\mathrm{kg}\;\mathrm{of}\;1\mathrm{RM}\cdot g\cdot \mathrm{distance}\right]/\mathrm{time})/\mathrm{body}\;\mathrm{mass}$	(3)
$1\mathrm{RM}\;=\;\mathrm{see}\;\mathrm{strength}\;\mathrm{score},\;g\;=\;9.81\;m/s^{2},\;\mathrm{distance}\;=\;\mathrm{Arm}\;\mathrm{length}-33\;\mathrm{cm},\;\mathrm{time}\;=\;1.5\;s.\;\mathrm{Arm}\;\mathrm{length}\;\mathrm{derived}\;\mathrm{from}$ (27)	
$\mathrm{DeadliftPowe}r_{1\mathrm{RM}}[W/\mathrm{kg}\;\mathrm{body}\;\mathrm{mass}]\;=\;(\left[\mathrm{kg}\;\mathrm{of}\;1\mathrm{RM}\cdot g\cdot \mathrm{lifting}\;\mathrm{height}\right]/s)/\mathrm{body}\;\mathrm{mass}$	(4)
1RM=seestrengthscore,g=9.81m/s,liftingheight:approximatedto1/4ofbodyheight	
$\mathrm{CoT}\;[W/\mathrm{kg}]\;=\;2.70\;+\;0.674\cdot e^{-18.24}\cdot \sin({\mathrm{incl}}^{\circ })\;\times \;v[m/s]$	(5)
CoT=Costoftransportofparalleltosurfacerunning (28)	
Relativeverticalmechanicalpower[W/kg]:Totalmass[kg]⋅v[m/s]⋅g⋅sin(°incl)/bodymass	(6)
Totalmass=bodymass+12.6kg(vest)+1kg(includingportablespiroergometer,clothesandshoes),v=2.222m/s	

#### 2.3.4 Severe-intensity muscular and cardiopulmonary work

Strength endurance was measured by weighted pullups (wPullups_rep_) and deadlifts (Deadlifts_rep_), respectively.

Participants should perform as many pullups as possible wearing a 12.6 kg weight vest from an overhand grip hanging position. Deadlifts were performed after the endurance test and approximately 20 min of rest, followed by a specific warm-up phase. Using an Olympic weight lifting bar (20 kg) with an additional 80 kg, participants were asked to lift the weight as many times as possible. A deadlift instruction video was distributed by the commands at least 2 weeks before the testing, and was shown again immediately before the deadlift test. Deadlifts were performed with wrist straps, and the test was terminated after repeated errors that could not be corrected, such as: participants not able to maintain a flat back, knee angle opens but hip angle unable to open as well, shoulders drop, rest more than 10 s in the lower position.

#### 2.3.5 Aerobic endurance

Endurance performance was measured by cardiopulmonary exercise testing (CPET) using a loaded treadmill ramp test protocol with 1° per min inclination increase and a constant speed of 8 km/h, after a 1 min warm up with 0° at 5.5 km/h. Participants wore a weight vest (12.6 kg). Pulmonary gas exchange was measured through a breath-by-breath spiroergometry system (MetaMax 3B-R2, Cortex Biophysics, Leipzig, Germany), with Hans Rudolph face-mask attached to the MetaMax 3B-R2. The system was turned on for at least 30 min to warm up, and then calibrated prior to every test day according to the manufacturer's recommendations. This involves first calibrating the gas analyzers by using a high precision gas (15% O_2_, 5% CO_2_ in N) and a 3-L syringe for volume flow calibration (both Cortex Biophysik GmbH, Leipzig, Germany), and verifying the calibration against ambient air. In prior research, the system showed excellent percentage errors of 1.95 ± 1.90 for respiratory gas exchange variables (32), and the additional loading with the weight west does not appear to impact the reliability of the MetaMax 3B-R2 system (33). Heart rate (HR), muscle oxygen saturation (SmO_2_) and total hemoglobin (THb) was recorded telemetrically using a belt-worn HR sensor (Polar H10, Polar Oy, Kempele, Finland) and two wearable near-infrared spectroscopy (NIRS) devices to monitor SmO_2_ and THb (Moxy, Fortiori Design LLC., Hutchinson, MN, USA).

Lactate (Lac) concentration was determined enzymatically using a photometer (Lactate Photometer Plus DP 110, Diaglobal, Berlin, Germany) from 10 μl of capillary blood, drawn from the ear lobe, immediately after test stop (0′ post) and five minutes after test stop (5′ post). Participants were instructed to voluntarily stop the test upon exhaustion by stepping to the non-moving sides of the treadmill (h/p Cosmos Pulsar, Nussdorf-Traunstein, Germany) and then sit down on a chair. Participants rated their perceived exertion using the Borg Scale6–20 (34).

Ventilatory thresholds (VT) 1 and 2, and $\dot{V}O_{2\max}$ were determined by a sport scientist who was highly experienced in CPET interpretation, using the steps described in the positional paper of the German working group “cardiopulmonary exercise testing” to ventilatory and metabolic (lactate) thresholds (35, 36). Thresholds of a sample of tests was cross checked and verified by a sports physician. VT1 and VT2 were determined visually using panels 5 (V-slope), 6, 1, and 9 of Wasserman's 2012 revised nine-panel-plot (37), using rolling averages of 30 s data from the ramp CPET.

In addition to measured inclination, the mechanical work of uphill running (28) (in Watts per kg body mass) wearing a weight west was calculated using the metabolic cost of level running (e.g., supporting body weight, braking and propelling body mass in the forward direction, maintaining balance) plus the vertical mechanical power, that is, the work to raise the body mass (m) against gravity (g). Net metabolic cost of transport of uphill and parallel to surface running (Equation 5) and the relative vertical mechanical power (Equation 6, Table 2) were added to obtain performance on the treadmill. P_max_ Incl was calculated using the maximal inclination angle the participant reached on the treadmill.

$\dot{V}O_{2}$ at 4° inclination (38) was used as a measure of the uphill running economy (URe4°).

Table 1 provides an overview of the fitness response variables.

#### 2.3.6 Training

Information about typical training volume and intensity was collected by a tailor-made questionnaire including questions about duration (in minutes per training session) and intensity (light, moderate, vigorous/high intense/maximal effort), as well as type of training (e.g., explosive, strength, endurance, high-intensity-interval, technical-tactical, sport-specific) from each of the past days of the week (morning and afternoon/evening) as anchor to minimize recall bias.

#### 2.3.7 Strain and recovery

Physical and mental strain and recovery was assessed using the six-item inventory “Beanspruchungs- und Erholungs-Monitoring Instrument” BEMI (39), with score points of overall strain and recovery, and the sub-scores strain, recovery, mental and physical balance resulting from z-transformed items. The questionnaire was normed using large samples of sports athletes, which means that a score of 0 is the strain and recovery of an average sport athlete, and negative scores meaning strain exceeding recovery.

All study data, including data from questionnaires and inventories, were collected and managed using REDCap electronic data capture tools (v13.7.3) (40) hosted at the Federal office of sport FOSPO, Magglingen, Switzerland.

### 2.4 Statistical analysis

Data were described by mean (*M*), standard deviation (*SD*), minimal and maximal values (Min, Max). Evidence for differences between groups and units was expressed as probability of group effect from Bayesian linear regression. All analysis were performed using RStudio (v2023.06.0) with R (v4.2.2) and (among others) the packages tidyverse (v2.0.0), brms [v2.20.4, see Bürkner (41)], FactoMineR (v2.9), and ggplot2 (v3.4.2).

Bayesian linear models, fitted using Markov Chain Monte Carlo (MCMC) sampling with 4 chains of 8,000 iterations and a warmup of 4,000, were used to estimate group effects. Priors over parameters were set as uniform location-scale t distributions (half Cauchy with a scale parameter that depends on the SD of the response variable), which can be considered a non-informative prior distribution. The brms default scale parameter *σ* is 2.5, and if *σ* is greater than 2.5, the Median Absolute Deviation (mad) of y is used as scale parameter. Posterior expectations with 95% credible interval (CI) were reported from models that showed no sign of non-convergence and instability (R-hat below 1.01 (42), and Effective Sample Size (ESS) greater than 1,000 (41)). The thresholds beyond which the group effect is considered non-negligible and large are 0.05 and 0.30 of the outcome's SD (similar to a “significant” result and a Cohen's *d* = 0.30). As measures to compare the null-model without the group/unit (m0) with the model containing the unit/group as fixed effect (m1), Bayes Factor (BF_10_) and leave-one-out cross-validation based on the posterior density (LOO) was used (43). BF_10_ is the ratio of the probabilities of the data under m1 and m0 and was computed by comparing the likelihoods under m1 and m0; a BF_10_ above 1 is an indication in favor of m1. In LOO, all models receive a difference score relative to the best model. Additionally, we report the Probability of Direction (pd), the proportion of the posterior distribution for the group coefficient lying entirely above or below zero. Within the scope of this article, we present those comparisons for which pd >90% (analogous to *α* = 0.05), which is considered non-negligible group effect.

Missing values—fully detailed in the Supplementary Material—were removed from the analysis. As these omissions resulted from equipment availability or malfunction factors unrelated to participants' fitness profiles, we do not anticipate systematic bias in our results.

## 3 Results

### 3.1 Participant characteristics

A total of 262 men—175 SOF candidates (CSOF), 69 SOF operators (TA), and 18 sports athletes (SA)—participated. Candidates were younger, lighter, and shorter than the overall mean; operators were older; athletes were tallest (Table 3).

**Table 3:** Participant characteristics, training volume, as well as strain and recovery indicators.

Characteristic	Overall	SOF candidates	Tactical athletes	Sports athletes
	*N* = 262	*N* = 175	*N* = 69	*N* = 18
Age				
Mean (SD)	28.5 (7.4)	26.4 (6.9)	34.0 (6.4)	28.1 (5.3)
Min—Max	18.0–52.0	18.0–50.0	23.0–52.0	19.0–37.0
Body mass				
Mean (SD)	80.8 (9.9)	79.1 (9.8)	83.7 (9.3)	85.7 (9.9)
Min—Max	57.2–111.4	57.2–104.3	63.7–110.9	68.7–111.4
Height				
Mean (SD)	180.1 (6.5)	179.7 (6.5)	180.2 (6.4)	183.8 (6.0)
Min—Max	164.2–201.0	164.2–198.5	168.0–201.0	168.1–193.2
BMI				
Mean (SD)	24.9 (2.5)	24.5 (2.6)	25.7 (2.2)	25.4 (2.6)
Min—Max	18.1–33.0	18.1–33.0	21.9–31.0	22.0–32.7
Total training hours per week				
Mean (SD)	7.3 (4.0)	6.9 (3.5)	8.5 (5.2)	9.5 (3.6)
Min—Max (Missing)	1.4–26.9 (30)	1.4–26.9 (5)	2.0–19.8 (14)	4.8–14.7 (11)
Total training hours per week in vigorous intensity				
Mean (SD)	1.79 (1.86)	1.75 (1.67)	1.73 (2.07)	3.39 (3.61)
Min—Max	0–12 (30)	0–10.8 (5)	0–12 (14)	0.4–10.3 (11)
BEMI overall				
Mean (SD)	19 (32)	25 (30)	6 (33)	22 (16)
Min—Max (Missing)	−88–92 (39)	−73–88 (17)	−88–92 (9)	8–42 (13)
BEMI psychological balance				
Mean (SD)	4 (14)	5 (14)	−1 (14)	5 (6)
Min—Max	−56–28	−56–28	−39–28	−3–11
BEMI physiological balance				
Mean (SD)	16 (24)	19 (23)	7 (25)	17 (17)
Min—Max	−49–72	−42–72	−49–72	−1–38
BEMI recovery				
Mean (SD)	9 (23)	15 (19)	−5 (26)	18 (17)
Min—Max	−68–61	−35–61	−68–52	1–47
BEMI strain				
Mean (SD)	−10 (16)	−10 (15)	−11 (18)	−4 (15)
Min—Max	−40–53	−40–49	−40–53	−27–11

### 3.2 Training volume

**Figure 2:**
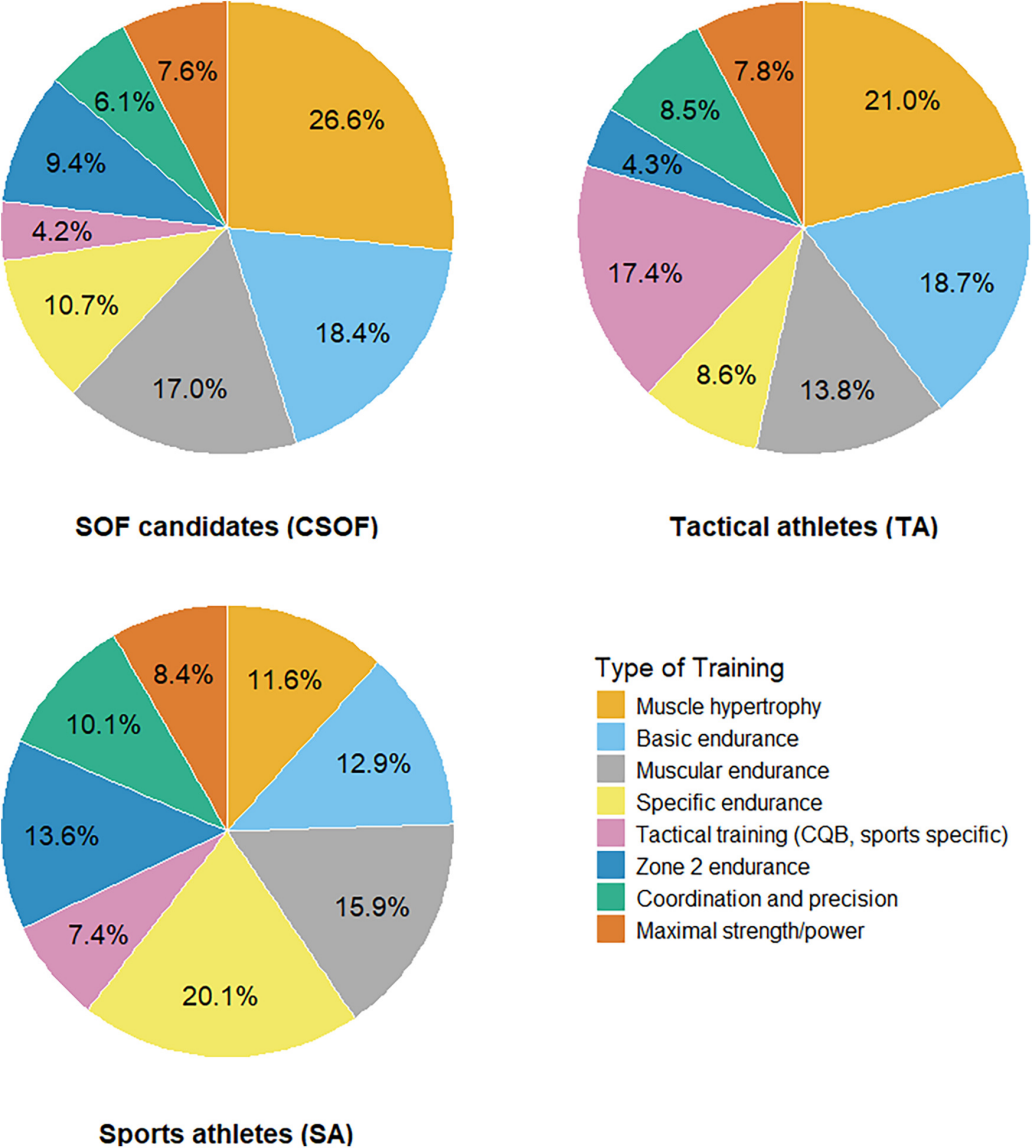
Proportions of type of training in special operation forces (SOF) candidates, tactical athletes and sports athletes (*n* = 236).

SA trained for an average of 9.5 h per week. TA had 8.5 h total hours of training per week, while CSOF trained 3 h less per week on average [95% CI (−0.4, 5.8), 95% probability of the group effect being non-negligible, Table 3]. Type of training per group is summarized in Figure 2, while Supplementary Table S2 displays the training hours per intensity level more in detail.

### 3.3 Strain and recovery

TA scored 16.4 points less [95% CI (−44.86, 11.12)] than SA in overall strain and recovery. On the BEMI subscale “recovery”, TA [Median = −23.07 points, 95% CI (−42.43, −4.20)], and within this group especially the MP SOF pro unit, felt less recovered [Median = −24.90 points, 95% CI (−47.87, −2.46), see Supplementary Table S3].

### 3.4 Fitness response variables

**Figure 3:**
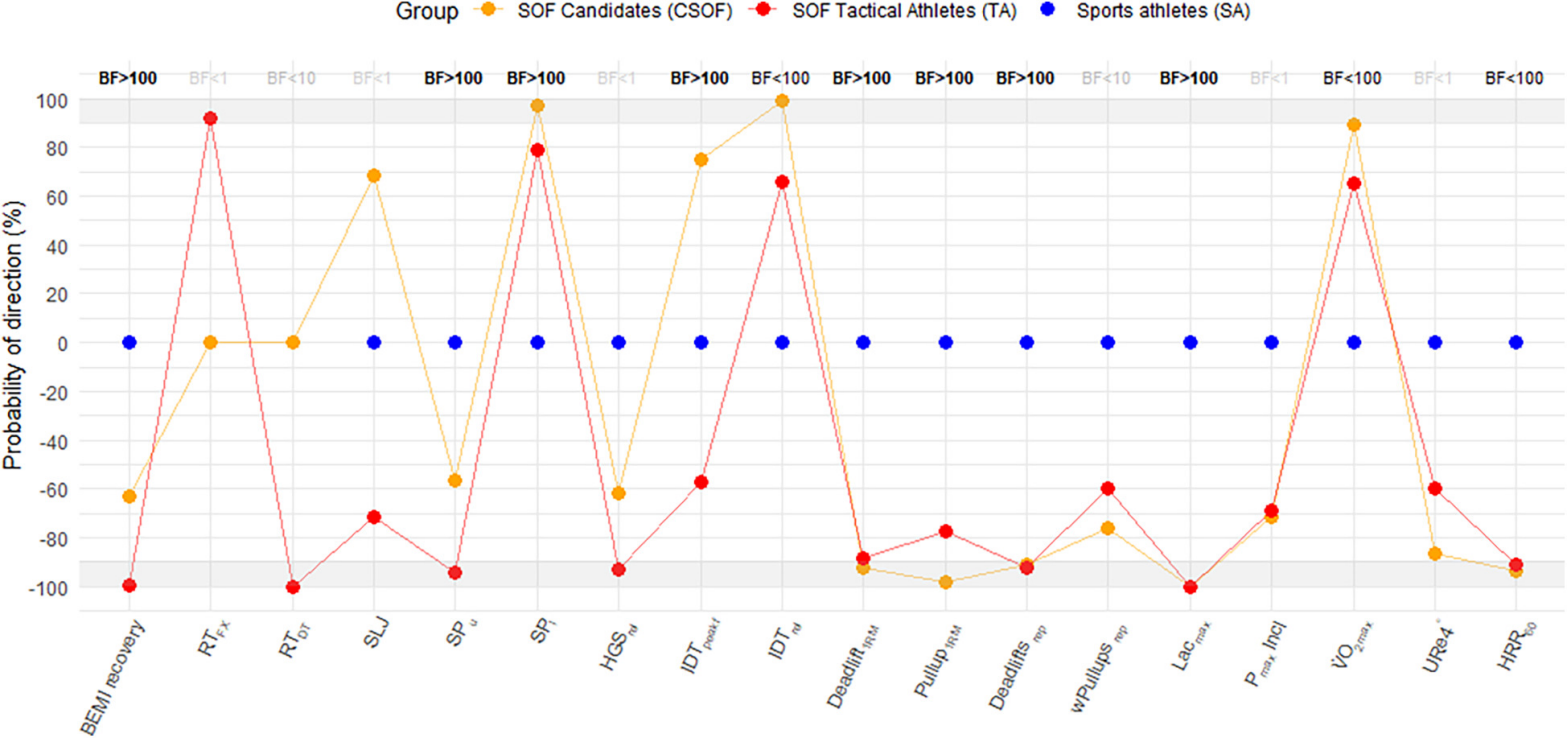
Evidence for group differences (SOF candidates and operators vs. sports athletes). Probability of Direction (pd): Each dot shows the pd—the certainty that the posterior distribution for the group effect is entirely on one side of zero. Dots above the horizontal zero line (positive pd) indicate evidence that the group outperformed the sports-athlete reference (blue dot at zero); dots below (negative pd) indicate lower performance. Grey shaded bands highlight pd ≥ 90% as “strong evidence” (analogous to *p* < 0.05). The blue dot at zero marks the sports-athlete baseline. Bayes Factor (BF₁₀): Numeric values in the upper panel show BF₁₀—the ratio of the probability of the data under the model including the group effect (m₁) vs. the null model (m₀), computed via marginal likelihoods. BF₁₀ < 1 indicates evidence against including the group effect; BF₁₀ 1–10 indicates weak evidence in favor; BF₁₀ 10–100 indicates strong evidence; and BF₁₀ > 100 indicates very strong or extreme evidence. RT_FX_: Mean reaction time FX/Fitlight; RT_DT_: Median reaction time Determination Test; SP_u,_ SP_l_: Striking power (upper body, lower body); HGS_rel_: Relative hand grip strength; IDT_pekf_: peak isometric force [N]; IDT_rel_: IDT peak kg relative to body mass; wPullups_rep_: weighted pullup repetitions; Lac_max_: Maximal blood lactate accumulation; P_max_ Incl: Performance at maximal inclination; P_VO2max_: Performance [W/kg body mass] at $\dot{V}O_{2max}$; URe4°: Uphill running economy [L/min at 4°]; HRR_60_: HR recovery 60 s after test stop.

Reaction Time: CSOF were faster than TA in the Determination Test [Median = −0.04 s, 95% CI (−0.02, −0.06)]. (Table 4; Figure 3, see supplementary Table S3 for details).

**Table 4:** Description of the fitness response variables.

Fitness dimension	Fitness response variable	Overall	SOF candidates	Tactical athletes	Sports athletes
		*N* = 262	*N* = 175	*N* = 69	*N* = 18
Executive function	Median DT Reaction time [s]				
	Mean (SD)	0.68 (0.06)	0.67 (0.06)	0.71 (0.06)	
	Min—Max (Missing)	0.51–0.85 (118)	0.51–0.82 (67)	0.61–0.85 (33)	NA (18)
	FX Reaction time [s]				
	Mean (SD)	2.13 (0.21)	2.14 (0.21)	2.08 (0.21)	
Power	Min—Max (Missing)	1.58–2.96 (76)	1.64–2.96 (20)	1.58–2.66 (38)	NA (18)
	SLJ [cm]				
	Mean (SD)	2.43 (0.19)	2.44 (0.19)	2.39 (0.19)	2.42 (0.24)
	Min—Max (Missing)	1.62–2.91 (1)	1.62–2.91	1.84–2.81	1.84–2.72 (1)
	Striking power lower body [f]				
	Mean (SD)	2,723 (917)	2,818 (886)	2,564 (875)	2,353 (1,273)
	Min—Max (Missing)	201–5,961 (7)	806–5,961 (3)	201–4,799 (1)	709–5,568 (3)
	Striking power upper body [f]				
	Mean (SD)	1,485 (646)	1,553 (659)	1,291 (534)	1,583 (811)
	Min—Max (Missing)	316–3,703 (7)	382–3,703 (3)	316–2,806 (1)	392–2,819 (3)
	Deadlifting power [W/kg body mass]				
	Mean (SD)	5.90 (1.74)	5.94 (1.79)	5.71 (1.41)	6.20 (2.38)
	Min—Max (Missing)	2.80–16.20 (10)	2.84–16.20 (1)	2.86–10.39 (5)	2.80–11.05 (4)
	Pullup power [W/kg body mass]				
Strength	Mean (SD)	4.83 (0.65)	4.80 (0.60)	4.86 (0.77)	4.94 (0.70)
	Min—Max (Missing)	3.61–7.24 (15)	3.61–6.24 (10)	3.71–7.24 (3)	4.02–6.33 (2)
	Handgrip strength [kg/kg body mass]				
	Mean (SD)	1.51 (0.23)	1.53 (0.23)	1.45 (0.19)	1.54 (0.26)
	Min—Max (Missing)	1.00–2.21 (2)	1.00–2.21 (1)	1.05–1.81	1.06–2.08 (1)
	Isometric deadlift test IDT [kg/kg body mass]				
	Mean (SD)	2.32 (0.44)	2.39 (0.43)	2.18 (0.40)	2.14 (0.51)
	Min—Max (Missing)	1.45–3.55 (8)	1.50–3.55	1.45–3.18 (7)	1.54–3.55 (1)
	Isometric deadlift test IDT peak force [N]				
	Mean (SD)	2,351 (353)	2,375 (364)	2,292 (312)	2,317 (372)
	Min—Max (Missing)	1,578–3,589 (8)	1,578–3,589	1,578–3,187 (7)	1,696–2,922 (1)
	Deadlift estimated 1RM [kg]				
	Mean (SD)	162 (52)	160 (54)	161 (40)	181 (78)
	Min—Max (Missing)	80–437 (10)	80–437 (1)	85–270 (5)	82–337 (4)
	Pullup estimated 1RM [kg]				
	Mean (SD)	118 (19)	115 (19)	122 (20)	126 (15)
Severe-intensity muscular and cardiopulmonary work	Min—Max (Missing)	75–177 (15)	75–177 (10)	85–161 (3)	101–158 (2)
	Deadlifts (reps) with 100 kg				
	Mean (SD)	20 (15)	20 (15)	19 (12)	25 (22)
	Min—Max (Missing)	0–101 (7)	0–101	0–51 (3)	2–71 (4)
	Weighed Pullups (reps) with 12.6 kg additional load				
	Mean (SD)	8.2 (5.0)	8.0 (4.7)	8.5 (5.6)	8.9 (5.4)
	Min—Max (Missing)	0.0–28.0 (2)	0.0–20.0	0.0–28.0	2.0–24.0 (2)
	Maximal treadmill inclination [°]				
	Mean (SD)	8.57 (1.49)	8.50 (1.44)	8.67 (1.70)	8.92 (1.03)
	Min—Max (Missing)	4.00–12.13	4.00–11.64	4.35–12.13	6.73–10.98
	Maximal lactate level [mmol/L]				
	Mean (SD)	14.0 (3.2)	13.9 (3.0)	14.8 (3.6)	11.3 (2.7)
	Min—Max (Missing)	5.4–23.3 (1)	5.4–22.0	6.8–23.3	6.5–16.8 (1)
	Performance at maximal inclination [W/kg body mass]				
	Mean (SD)	6.71 (0.62)	6.70 (0.61)	6.70 (0.67)	6.79 (0.45)
	Min—Max (Missing)	5.00–8.14 (1)	5.00–8.04 (1)	5.08–8.14	5.91–7.76
Aerobic endurance	$\dot{V}O_{2\max}$ [ml/kg/min]				
	Mean (SD)	54 (7)	55 (7)	53 (7)	53 (6)
	Min—Max (Missing)	35–79 (1)	35–72 (1)	38–79	44–64
	Performance at $\dot{V}O_{2\max}$ [W/kg body mass]				
	Mean (SD)	6.52 (0.62)	6.51 (0.61)	6.52 (0.68)	6.60 (0.47)
	Min—Max (Missing)	4.86–8.05 (1)	4.86–7.83 (1)	5.02–8.05	5.83–7.55
	Performance at VT2 [W/kg body mass]				
	Mean (SD)	5.94 (0.61)	5.93 (0.62)	5.95 (0.60)	6.04 (0.59)
	Min—Max (Missing)	4.37–7.54 (8)	4.46–7.37 (7)	4.70–7.54 (1)	4.37–7.06
	Percentage of max treadmill inclination at VT2				
	Mean (SD)	80 (11)	79 (10)	80 (10)	79 (15)
	Min—Max (Missing)	28–99 (8)	38–98 (7)	58–99 (1)	28–90
	Percentage of max treadmill inclination at VT1				
	Mean (SD)	21 (13)	19 (11)	25 (14)	24 (15)
	Min—Max	0–65	0–63	7–65	7–52
	Running economy at 4° [L/min]				
	Mean (SD)	3.30 (0.40)	3.27 (0.35)	3.35 (0.49)	3.37 (0.41)
	Min—Max	2.56–5.04	2.60–4.25	2.56–5.04	2.67–4.22
	HR recovery 60 s after test stop [bpm]				
	Mean (SD)	−29 (8)	−29 (7)	−29 (8)	−32 (7)
	Min—Max (Missing)	−44 (20)	−42 (9)	−44 (9)	−21 (2)

Power: TA had less striking power than the SA group [Median = −285.61 f, 95% CI (−644.18, 61.34)]; standing long jumps were comparable (239–244 cm).

Strength: Compared to SA, CSOF lifted more kg per kg body mass [Median = 0.26 kg/kg body mass, 95% CI (0.04, 0.47)]. With regard to 1RM in the pullup discipline, CSOF had less 1RM than SA [Median = −10.77 kg, 95% CI (−20.43, −1.19)].

Severe-intensity work: CSOF [Median = −5.38, 95% CI (−13.23, 2.51)] and TA [Median = −6.34 reps, 95% CI (−14.73, 2.17)] lifted the 100 kg fewer times than SA. SA accumulated less capillary blood lactate than CSOF [Median = 2.63 mmol/L, 95% CI (1.05, 4.22)], and TA [Median = 3.48 mmol/L, 95% CI (1.78, 5.14)].

Aerobic endurance: CSOF reached higher $\dot{V}$O_2max_ [Median = 2.07 ml/kg/min, 95% CI (−1.27, 5.39)] compared to SA. In turn, SA had higher HR recovery 60 s after the ramped CPET than CSOF and TA.

## 4 Discussion

The present study describes a multidimensional fitness profile for Swiss SOF operators and candidates—anchored in practitioner-derived KPIs—and presents evidence for both differences and non-differences relative to sports athletes. This approach aligns with the multi-layered validity framework of James et al. (44), which evaluates test suitability across measurement quality, decision-making relevance, and organizational feasibility. A key distinction from Eisinger et al. (10) lies in our three-fold test-construction strategy: (1) anchoring each assessment in practitioner-defined CSFs translated into sport-science KPIs; (2) leveraging established protocols (e.g., standing long jump, $\dot{V}O_{2\max}$ testing, hand-grip dynamometry) to draw on existing normative data; and (3)—critically—ensuring face validity by selecting or adapting tests that SOF operators and commands immediately recognize as tests that capture “fit for” their selection, successful operational readiness and training requirements. The scientific rigor, combined with new test elements and high acceptance makes our battery actionable for elite SOF contexts.

SOF candidates in our sample devoted nearly half their training to hypertrophy and basic endurance, whereas sports athletes emphasized discipline-specific endurance. Tactical athletes' year-round balance of strength, endurance, and tactical drills underscores the importance of training programs that address block periodization model (45).

Professional tactical athletes are hardly recovered, reflecting the nature of their occupation. Without training periodization, they always need to be fully operational ready for whatever mission (3, 4, 46). Given that shift work in police units may negatively impact stress and recovery (47), and tactical athletes were generally less recovered, this could partially explain the faster reaction times of SOF candidates in the computer-based Determination Test. However, in a reaction time test simulating close-quarters combat—incorporating visual scanning, decision-making, and real gun handling—tactical athletes reacted faster than candidates. Notably, the most experienced operators from the professional MP SOF unit outperformed all other participants by 0.138 s, despite being the least recovered unit according to the BEMI. This reaction test appears to capture a critical performance demand for SOF tactical athletes, as it closely reflects real-world tactical scenarios, including firearm handling.

Domain-specific trade-offs emerged in the physical fitness tests: Sports athletes led in raw striking power and repeated-lift endurance, whereas SOF personnel maintained robust lower-body power and relative deadlift strength. These findings highlight how training emphases shape performance and necessitate personalized programming.

Standing long-jump distances align with Norwegian (13), Finnish (48), Dutch SOF (49) and U.S. Navy SEAL (50) units (The operators achieved standing long jump distances between 2.32 and 2.50 m; in comparison, NFL Combine athletes demonstrate an impressive average of 2.92 m (51), while the current world record, held by Norwegian Arne Tvervaag, stands at 3.71 m); grip strength, weighted pull-up, deadlift, and $\dot{V}$O_2max_ values similarly mirror published norms (8, 13, 46, 50, 59), validating our battery's external relevance.

### 4.1 Practical applications

Our reference values allow SOF fitness trainers and commanders to identify individual deficits—whether in power, endurance, or executive functions—and prescribe truly individualized interventions. Units might adopt severe-endurance benchmarks typical of competitive boxers or wrestlers for high-intensity work blocks or use the decathlete's well-rounded profile to guide balanced development across explosive power, strength, and aerobic capacity. The battery's ecological and face validity supports its integration into both selection pipelines and routine readiness assessments.

### 4.2 Limitations & future directions

This study is observational, and merely describing and comparing groups does not directly support candidates or tactical athletes in their efforts to prepare for the selection process or maintain operational fitness for missions. Further, our sample is not homogeneous by age—a factor that may confound predictive models. We observed that younger SOF candidates outperformed operators in domains susceptible to age effects—such as computerized reaction speed, standing long jump explosiveness, and peak isometric force—reflecting well-documented advantages in processing speed and explosive power among younger adults. However, our experienced operators consistently sustain high-level fitness across these same measures despite continuous training loads and greater age, setting a practical benchmark: candidates must match or exceed operator performance in any age-sensitive domain to demonstrate true operational readiness. Other limitations include: Our sports-athlete cohort was a convenience sample and smaller than SOF groups. Fixed vest loads (12.6 kg) impose different relative demands across body masses; future work should explore individualized vest weights (e.g., percentage of body mass). The use of Fitlight® with a simulation handgun in tactical scenarios is novel; subsequent research should assess its test–retest reliability and criterion validity against gold-standard motion-capture or high-speed video systems in SOF populations. We did not employ structured consensus methods; future efforts should apply approaches such as the Delphi method or nominal group technique to refine and validate CSFs, thereby enhancing KPI selection's methodological rigor.

## 5 Conclusions

In this study, we defined and operationalized a multidimensional fitness profile for tactical athletes by translating practitioner-derived Critical Success Factors for basic SOF training and missions into sport-science Key Performance Indicators and deploying a high-face-validity test battery under operational load—covering (a) muscular impulse production (power), (b) maximal strength, (c) severe-intensity muscular and cardiopulmonary work capacity, (d) aerobic endurance, and (e) reaction time as part of executive functioning. We established initial reference values and revealed both strong parallels and task-specific distinctions in fitness profiles: Swiss personnel perform on par with international SOF units and high-level athletes, despite the continuous operational demands that limit recovery.

This test battery provides a comprehensive fitness profile for SOF personnel, allowing individuals to identify and complement potential weaknesses. Such profiling, including training volume, may inform individualized training recommendations and future research aimed at predicting high-performing SOF operators or elite athletes in specific sports.

## Data Availability

The datasets presented in this article are not readily available because the raw data are not transferable due to their military classification. Requests to access the datasets should be directed to alain.doessegger@baspo.admin.ch.
