# Supplementary material for: Fitness profile and training of Special Operation Forces: a comparison with sports athletes
Source: Front Sports Act Living. 2025 May 21;7:1594714. doi: 10.3389/fspor.2025.1594714 (PMC12133949; doi:10.3389/fspor.2025.1594714)
Supplement: Supplementary file 1 [file Datasheet1.docx]

Supplementary Material

# Supplementary Data

- Table S1
- Table S2
- Table S3
- Missing values explanation

# Supplementary Figures and Tables

## Supplementary Tables

**Supplementary Table S1.**

Participants recruited for the study: Professional, militia and support units, as well as sports athletes as a comparison group.

| Group | Category | Unit |
| --- | --- | --- |
| SOF candidates (CSOF) | Professional | Police SOF |
|  | Militia | Grenadier |
|  | Militia | Para-recon |
|  | Militia | MP Grenadier |
| Tactical athletes (SOF operators) (TA) | Professional | MP SOF pro |
|  | Professional | Police SOF |
|  | Support | SOFA |
|  | Militia | Police SOF |
| Sports athletes (SA) |  | Sports athletes |

Notes. SOF: Special Operation Forces; Grenadier: Swiss Special Forces Command operators; Para-recon: Parachute Reconnaissance operators of the Swiss Special Forces Command; MP Grenadier: Military Police grenadiers; MP SOF pro: Professional tier 1 Swiss Special Forces Command operators; SOFA: Military VIP close protection and special vehicle driver specialist.


**Supplementary Table S2.**

*Training hours in specific intensities of Swiss SOF candidates, sports and tactical athletes (n = 236)*

|  |  | **n** | **Mean** | **SD** | **Maximum h** |
| --- | --- | --- | --- | --- | --- |
| Light intensity training h | SOF candidates | 174 | 2.11 | 2.161 | 14.83 |
|  | Sports athletes | 7 | 2.62 | 1.900 | 5.58 |
|  | Tactical athletes | 55 | 1.74 | 2.142 | 10.50 |
| Moderate intensity training h | SOF candidates | 174 | 2.94 | 2.222 | 16.25 |
|  | Sports athletes | 7 | 3.49 | 1.978 | 6.00 |
|  | Tactical athletes | 55 | 5.04 | 4.779 | 17.33 |
| Vigorous intensity training h | SOF candidates | 174 | 1.73 | 1.662 | 10.83 |
|  | Sports athletes | 7 | 3.39 | 3.610 | 10.33 |
|  | Tactical athletes | 55 | 1.73 | 2.068 | 12.00 |
| Total training h | SOF candidates | 174 | 6.78 | 3.548 | 26.92 |
|  | Sports athletes | 7 | 9.50 | 3.623 | 14.67 |
|  | Tactical athletes | 55 | 8.52 | 5.154 | 19.75 |
|  | | | | | |

**Supplementary Table S3.**

Results of the most important fitness response variables, stratified by SOF candidates, tactical athletes (currently active operators) and sports athletes. Evidence for group effect is printed in bold (for effects larger than 90%). The probability of a noteworthy (non-negligible) effect of the group and the unit, as well as the probability of the effect being large (in parentheses), is indicated.

| Response variable | SOF candidates (CSOF) | SOF operators/tactical athletes (TA) | Sports athletes (SA) | Probability of non-negligible group effect | Probability of non-negligible effects on unit level |
| --- | --- | --- | --- | --- | --- |
|  | *N = 175* | *N = 69* | *N = 18* | *P = Probability for non-negligible effect and the probability for the effect being large (in parenthesis)* | *P = Probability for non-negligible effect and the probability for the effect being large (in parenthesis), compared to sports athletes (Reference group = SA if not indicated otherwise)* |
| BEMI recovery [score] | 15 (19) | **-5 (26)** | 18 (17) | TA vs SA: 98.9% P for less recovery (95.2%) | MP SOF: 98.1% P for less recovery (93.9%) |
| Mean reaction time FX/Fitlight (RT_FX_) [s] | 2.14 (0.21) | 2.08 (0.21) | NA |  | MP SOF: 99.0% P for faster RT (97.7%) compared to Grenadier |
| Median reaction time DT (RT_DT_) [s] | **0.67 (0.06)** | 0.71 (0.06) | NA | C vs TA: 99.9% P for faster RT (97.5%) | All units except MP Grenadier: >99.2% P for slower RT (>95.1%) compared to Grenadier |
| Standing longjump (SLJ) [m] | 2.44 (0.19) | 2.39 (0.19) | 2.42 (0.24) |  | Grenadier: 92.0% P for more distance (69.4%); SOFA: 99.2% P for less distance (93.5%) |
| Upper body striking power (SP_u_) [f] | 1553 (659) | **1291 (534)** | 1583 (811) | TA vs SA: 91.9% P for less power (68.8%) | Grenadier: 91.0% P for more power (68.4%) |
| Lower body striking power (SP_l_) [f] | **2818 (886)** | 2564 (875) | 2353 (1,273) | C vs SA: 95.6% P for more power (78.6%) | Grenadier: 97.0% P for more power (86.2%); Police SOF: 96.1% P for more power (80.2%) |
| Handgrip (HG) relative grip strength (HG_rel_) | 1.53 (0.23) | 1.45 (0.19) | 1.54 (0.26) |  | Grenadier: 90.9% P for more HG (66.9%) |
| IDT peak force (IDTpeakf) [N] | 2375 (364) | 2292 (312) | 2317 (372) |  |  |
| IDT peak kg relative to body mass (IDT_rel_) | **2.39 (0.43)** | 2.18 (0.40) | 2.14 (0.51) | C vs SA: 98.2% P for more strength (86.7%) | Grenadier: 99.7% P for more strength (96.9%); Para-recon: 99.6% P for more strength (96.3%) |
| One repetition maximum deadlift (Deadlift_1RM_) [kg] | 160 (54) | 161 (40) | 181 (78) | C vs SA: 89.0% P for less strength (62.6%); TA vs SA: 84.8% P for less strength (56.6%) | Grenadier, MP Grenadier, Para-recon, and SOFA: >96.3% P for less strength (>81.7%) |
| One repetition maximum pullup (Pullup_1RM_) [kg] | **115 (19)** | 122 (20) | 126 (15) | C vs SA: 97.8% P for less strength (85.3%) | Grenadier, MP Grenadier, Para-recon, and SOFA: >98.8% P for less strength (>94.8%); MP SOF pro: 57% P for more strength (27.6%) |
| Repetitions deadlift (Deadlifts_rep_) | 20 (15) | **19 (12)** | 25 (22) |  | Grenadier, MP Grenadier, Para-recon, and SOFA : >93% P for less strength (>74.4%) |
| Repetitions weighted pullups (wPullups_rep_) | 8.0 (4.7) | 8.5 (5.6) | 8.9 (5.4) |  | SOFA: 100% P for less reps (99.6%) |
| Maximal blood lactate accumulation (Lac_max_) [mmol/L] | **13.9 (3.0)** | **14.8 (3.6)** | 11.3 (2.7) | C vs SA: 99.9% P for more lactate (98.0%); TA vs SA: 100% P for more lactate (99.9%) | Police SOF: 100% P for more (100%); Grenadier: 99.5% P for more (95.5%); MP SOF pro: 99.4% P for more lactate (95.6%) |
| Performance at maximal inclination (P_max_ Incl) [W/kg] | 6.70 (0.61) | 6.70 (0.67) | 6.79 (0.45) |  | Para-recon: 93.4% P for more max inclination (71.3%); SOFA: 100% P for less max inclination (100%) |
| $\dot{V}$O_2max_ [ml/kg/min] | 55 (7) | 53 (7) | 53 (6) | C vs SA: 84.8% P for higher aerobic capacity (50.6%) | Para-recon: 100% P more (99.5%); Grenadier: 99.3% P more (92.9%); MP Gren: 92.8% P more (78.3%); SOFA: 99.1% P less aerobic capacity (92.3%) |
| Uphill running economy (URe4°) [L/min] | 3.27 (0.35) | 3.35 (0.49) | 3.37 (0.41) |  | Para-recon: 96.4% P for more economic running (82.6%) |
| HR recovery 60 s after test stop (HRR_60_) [bpm] | **-29 (7)** | -29 (8) | -32 (7) | C vs SA: 91.0% P for less recovery (64.9%); TA vs SA: 87.8% P for less HR recovery (60.4%) | SOFA: 99.9% P for less (98.5%); Police SOF: 98.2% P for less (86.9%); MP SOF pro: 93.3% P for less HR recovery (77.6%) |

# Missing values explanations

Explanation for missing values:

- BEMI had missing values due to non-compliance in filling out the online questionnaire

- Missing values in reaction time was due to non-availability of the Vienna Test System and malfunctioning of the Fitlight® system, that could only be solved by changing to a newer version of the sensors.

- SP missing values were due to a severe wear-out of the striking device and the need for replacement.

- IDT missing values were due to the non-availability of the device at the early stages of the study.

- Some participants had shoulder pain or back pain at the test date and decided not to perform the pullups or the deadlifts.

- EOD unit did not perform the treadmill test due to time constrains, instead they were tested in the IFT 30-15 test (data not shown).

- The treadmill test had missing values due to system failures (e.g., HR sensor) or non-detectable VT2.
